# The impact of *Babesia ovis*-infected *Rhipicephalus bursa* larvae on the severity of babesiosis in sheep

**DOI:** 10.3389/fcimb.2025.1544775

**Published:** 2025-02-20

**Authors:** Sezayi Ozubek, Mehmet Can Ulucesme, Onur Ceylan, Ferda Sevinc, Munir Aktas

**Affiliations:** ^1^ Department of Parasitology, Faculty of Veterinary Medicine, University of Firat, Elazig, Türkiye; ^2^ Department of Parasitology, Faculty of Veterinary Medicine, University of Selcuk, Konya, Türkiye

**Keywords:** *Babesia ovis*, experimental infestation, ovine babesiosis, *Rhipicephalus bursa*, transmission dynamics

## Abstract

Ovine babesiosis, caused by *Babesia ovis*, is a significant tick-borne disease affecting sheep globally, with severe economic implications for sheep farming, particularly in Türkiye. *Babesia ovis* is transmitted exclusively by adult *Rhipicephalus bursa* ticks, but the potential role of infected larval stages in modulating disease severity has remained unclear. This study investigated whether infestation with *B. ovis*-infected *R. bursa* larvae reduces the severity of babesiosis following subsequent exposure to infected adult ticks. Three experimental sheep were infested with *B. ovis*-infected larvae, while three control sheep were infested with *Babesia*-free larvae. Both groups were subsequently exposed to *B. ovis*-infected adult *R. bursa*. Daily clinical, molecular, and serological monitoring revealed no clinical signs of babesiosis or *B. ovis* infection following larval infestation. However, all sheep developed severe clinical babesiosis after exposure to infected adult ticks. No significant differences in disease severity, parasitemia levels, or clinical outcomes were observed between the experimental and control groups, indicating that larval infestation does not confer protection or lead to milder disease courses. These findings confirm the exclusive role of adult *R. bursa* in *B. ovis* transmission and emphasize the critical need for vector control strategies targeting adult tick populations during peak activity. This study highlights the importance of understanding stage-specific transmission barriers and their implications for vector-borne disease management. Future research should explore the molecular mechanisms limiting pathogen transmission by immature ticks and investigate comparative transmission dynamics across *Babesia* species to inform targeted control interventions.

## Introduction

Ovine babesiosis, caused by several *Babesia* species, is a significant tick-borne disease that affects sheep. The primary *Babesia* species known to infect sheep include *Babesia ovis*, *B. motasi*, *B. crassa*, *B. taylori*, *B. foliate*, *Babesia* sp. Xinjiang, *B. motasi* Lintanensis, and *B. motasi* Hebeinensis (Ceylan et al., 2021; Schnittger et al., 2022). Despite the presence of around eight species, *B. ovis* is recognized as the most pathogenic and potentially lethal species for sheep (Ceylan et al., 2021; Firat et al., 2024). It has been reported in several countries, including Türkiye (Ceylan and Sevinc, 2020; Ulucesme et al., 2023; Bozan et al., 2024), Pakistan (Iqbal et al., 2011), Iran (Gh et al., 2020), Spain (Ferrer et al., 1998), Portugal (Horta et al., 2014), Egypt (Hussein et al., 2017), Palestine (Azmi et al., 2016), Israel (Pipano, 2022), Greece (Theodoropoulos et al., 2006), Italy (Savini et al., 1999), Nigeria (Adewumi et al., 2022), Uganda (Tumwebaze et al., 2020), Algeria (Aouadi et al., 2017), and Tunisia (Rjeibi et al., 2016). However, in many of these regions, little is known about the mortality rates or economic impact of the disease on sheep farming. As a result, ovine babesiosis is often classified as a neglected disease, particularly when compared to bovine babesiosis (Ozubek et al., 2020).

In Türkiye, *B. ovis* is a significant concern in sheep farming, where the disease can result in mortality rates as high as 50% (Aktas et al., 2007; Ceylan et al., 2021). The economic implications of this disease are substantial, including costs related to medication and losses from sheep mortalities (Sevinc et al., 2007; Sevinç and Xuenan, 2015). *Babesia ovis* is transmitted by the two-host tick *Rhipicephalus bursa*, with the adult stage of the tick serving as the primary vector for *B. ovis* transmission (Altay et al., 2008; Erster et al., 2016; Antunes et al., 2018). The larvae of *R. bursa* remain inactive during the hot, dry summer months and begin to climb plants in early October, peaking in activity by December and subsiding by February. Larvae and nymphs enter a state of diapause during summer and winter, respectively, and adult ticks become active once temperatures exceed 18°C during the day and 12°C at night. There is approximately a six-month interval between the host-seeking activities of immature and mature ticks (Yeruham, 1995; Yeruham et al., 1998, 2000). This natural temporal separation between immature and adult ticks is relevant for understanding the transmission dynamics of *B. ovis*, particularly in assessing whether infestation with *B. ovis*-infected larvae has any impact on the severity of subsequent infections caused by adult ticks.

While *R. bursa* larvae and nymphs can be infected with *B. ovis*, these immature stages are incapable of transmitting the pathogen. Transmission occurs exclusively during the adult stage of the tick (Erster et al., 2016; Firat et al., 2024). Despite this, some studies suggest that infestation with *B. ovis*-infected larvae may have an immunizing effect, leading to milder infections when adult ticks transmit the pathogen later in life (Yeruham, 1995; Yeruham et al., 1998; Erster et al., 2016). However, the clinical and immunological implications of this interaction remain poorly understood, particularly regarding whether prior exposure to infected larvae influences the severity or resilience of sheep during later infections.

This study aimed to investigate whether infestation with *B. ovis*-infected *R. bursa* larvae alters the clinical course of babesiosis in sheep, contributing to the broader understanding of ovine babesiosis. By focusing on this aspect, our research goes beyond merely demonstrating the inability of larvae to directly transmit the pathogen, instead exploring their potential indirect effects on host-pathogen dynamics. Understanding these interactions is critical for improving control strategies, especially in endemic regions like Türkiye, where the economic impact of babesiosis on sheep farming is significant. Our findings aim to provide novel insights into the clinical outcomes of larval feeding, addressing critical gaps in the global understanding of vector-host interactions in babesiosis.

## Methods

### Ethics statement

This study was carried out according to the regulations of animal and welfare issued by the Turkish legislation for the protection of animals. All animal experiments were approved by the Firat University, Animal Experiment Ethic Committee, protocol number 2023/12-05. To minimize stress during the acclimatization period, all sheep were housed individually in a quiet and temperature-controlled environment, with daily health checks performed by trained personnel.

### Study animals and experimental design

**Figure 1:**
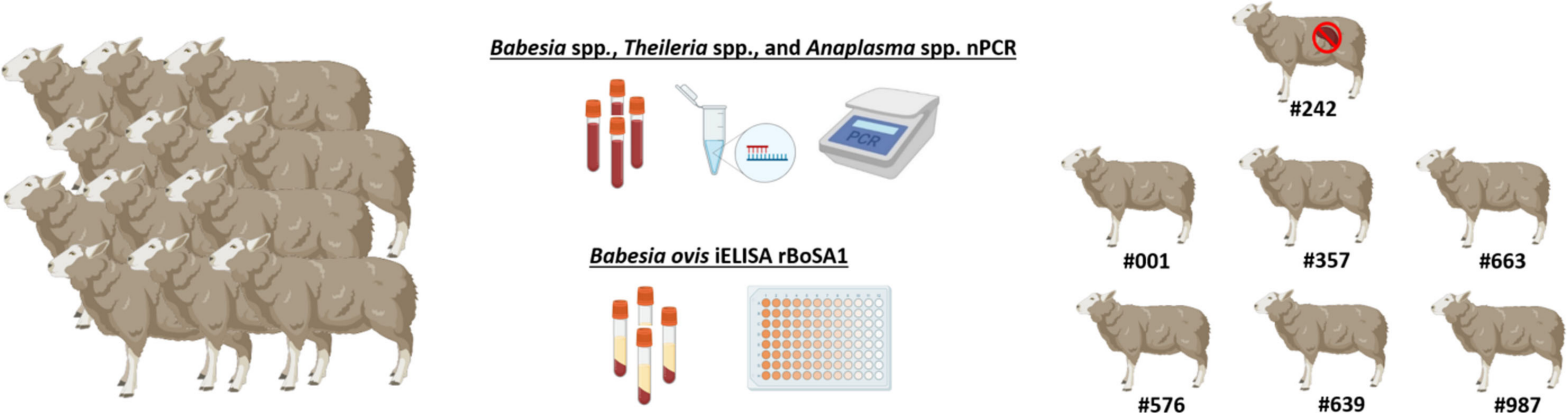
Selection of sheep for experimental infection study. All sheep were tested for *Babesia* spp., *Theileria* spp., and *Anaplasma* spp. using nPCR and screened for *B*. *ovis* using iELISA. Only those negative in both tests were included in the study. Figure was created using Biorender.com.

Seven clinically healthy Akkaraman sheep, aged between 5 and 8 months, were included in the study. These animals were selected after a rigorous screening process to confirm their freedom from infections with *Babesia*, *Theileria*, and *Anaplasma* species. Molecular analyses were performed using nested PCR (nPCR), targeting conserved genomic regions specific to these pathogens. Primer pairs used included Ec9/Ec12A (Kawahara et al., 2006) and 16S8FE/B-GA1B (Bekker et al., 2002) for *Anaplasma* spp., Nbab1F/Nbab1R (Oosthuizen et al., 2008) and RLBF2/RLBR2 (Georges et al., 2001) for *Babesia*/*Theileria* spp (**Supplementary Table 1**). Blood samples testing negative in these assays were further analyzed for the presence of antibodies against *B. ovis* using an in-house developed indirect ELISA (iELISA) targeting the recombinant *B. ovis* Secreted Antigen-1 (rBoSA1), as described by Sevinc et al. (2015). The rBoSA1 protein was expressed and purified using recombinant DNA technology in *Escherichia coli*. For the assay, 96-well ELISA microplates were coated with 50 µl of rBoSA1 at a concentration of 2 μg/ml, prepared in carbonate-bicarbonate buffer (pH=9.6). After an overnight incubation at 4°C, the plates were washed and blocked to prevent non-specific binding. Sheep sera were diluted at 1:100 in blocking buffer and incubated in the coated wells, followed by the addition of horseradish peroxidase-conjugated secondary antibodies (peroxidase-conjugated anti-sheep IgG; Sigma-Aldrich). The reaction was developed using a chromogenic substrate, and optical densities (OD) were measured at 450 nm. The cutoff value was calculated as the mean OD of negative control sera plus three times the standard deviation, ensuring accurate differentiation between positive and negative samples (Sevinc et al., 2015; Firat et al., 2024). Due to the lack of commercially available ELISA kits for ovine *Theileria* and *Anaplasma* species, no serological testing was performed for these pathogens. Based on the molecular and serological analyses, seven sheep were confirmed to be free of *Babesia*, *Theileria*, and *Anaplasma* infections. These animals were then transported to the Elazig Veterinary Control Institute Directorate, where they were housed individually in tick-free and biosecure compartments. The sheep were provided with *ad libitum* access to food and water throughout the study period. To ensure acclimatization and minimize stress, the animals were allowed to rest for seven days before the experiments began (**Figure 1**).

The sheep were assigned to two experimental groups. One sheep (#242) was designated for the production of *B. ovis*-infected *R. bursa* larvae, while the remaining six were randomly divided into an experimental group (#001, #357, #663) and a control group (#576, #639, #987). The sample size, consisting of three animals per group, was determined in accordance with ethical guidelines, emphasizing the principle of using the minimum number of animals necessary to achieve valid scientific results. This approach aligns with previous experimental infection studies involving *Babesia* species, where three animals per group have been widely adopted as standard practice (Alzan et al., 2021; Bastos et al., 2022; Silva et al., 2023; Firat et al., 2024).

### Production of *B. ovis*-infected *R. bursa* larvae

To produce *B. ovis*-infected *R. bursa* larvae, the designated sheep (#242) underwent splenectomy. Splenectomy is a widely adopted method in *Babesia* infection studies as it suppresses the host’s immune clearance mechanisms, thereby facilitating higher parasitemia and improving the efficiency of vector infection (Sevinc et al., 2007). Following the surgical operation, the splenectomized sheep was intravenously inoculated with 15 mL of cryopreserved *B. ovis* Alacakaya stabilate containing 5% parasitized erythrocytes (PPE). This stabilate, derived from a naturally infected sheep, had been purified through transovarial passage in *R. bursa* ticks to eliminate co-infections. It was cryopreserved with 10% dimethyl sulfoxide (DMSO) as a cryoprotectant and stored in liquid nitrogen at -196°C until use.

**Figure 2:**
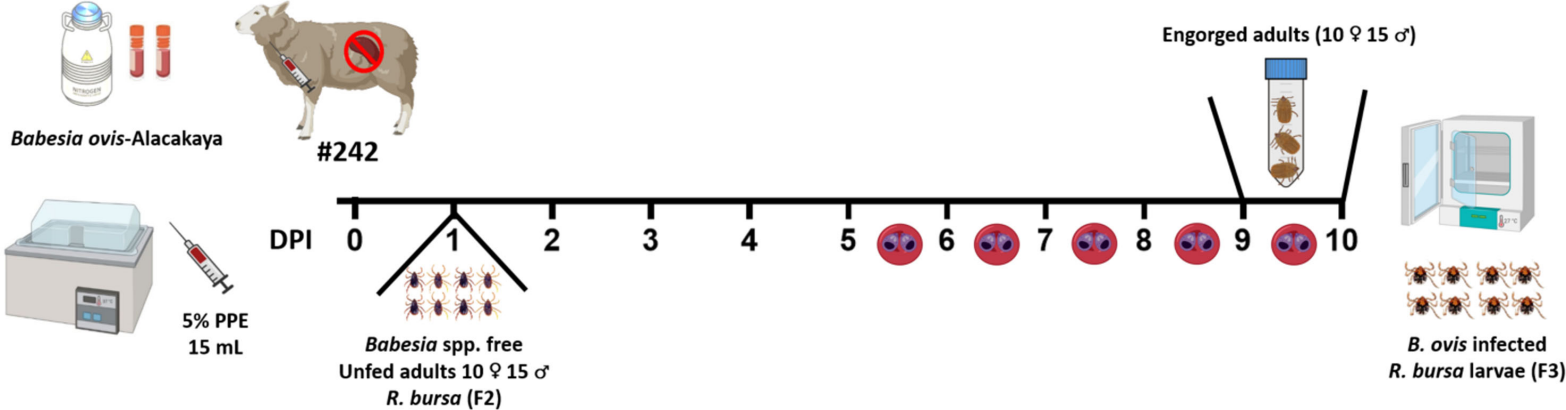
Experimental timeline for obtaining *B*. *ovis*-infected *R. bursa* larvae. Sheep #242 was inoculated with the *B*. *ovis*-Alacakaya stabilate on day 0. Unfed adult ticks (10 ♂ and 15 ♀) from a *Babesia*-free *R. bursa* (F2 generation) were placed on the sheep on DPI 1. The timeline shows daily post-infection days (DPI) up to day 10, highlighting days 5 to 10 where parasitemia was observed. Engorged females were collected on DPI 9 and 10 and placed in an incubator for breeding to produce *B*. *ovis*-infected (F3 generation) larvae. Figure was created using Biorender.com.

On the first day post-inoculation (DPI 1), 25 adult *R. bursa* ticks (10 females and 15 males), reared in a laboratory colony (Firat et al., 2024; Ulucesme et al., 2024), were attached to the splenectomized sheep using EVA foam feeding capsules (Almazán et al., 2018). These capsules, affixed to the shaved thoracic region of the sheep, allowed for controlled tick feeding. Parasitemia in the splenectomized sheep was monitored daily, and engorged female ticks were collected on days 9 and 10 post-infection (DPI 9 and 10). The collected ticks were incubated under controlled conditions (27°C and 85% relative humidity) to produce larvae. The larvae (500 larvae from each female tick) were tested using nPCR to confirm *B. ovis* infection, and only those confirmed to be positive were used for subsequent infestations (**Figure 2**).

### Experimental infestation and monitoring

At the beginning of the experiment, sheep in the experimental group (#001, #357, #663) were infested with 0.1 grams of *R. bursa* larvae infected with *B. ovis*, while those in the control group (#576, #639, #987) were infested with the same quantity of *Babesia*-free *R. bursa* larvae (Firat et al., 2024; Ulucesme et al., 2024). This initial infestation ensured uniform exposure across both groups, enabling the evaluation of the effects of *B. ovis*-infected larvae compared to uninfected larvae. Following infestation, the larvae were monitored daily, and engorged nymphs were collected from each sheep and placed in separate plastic containers for incubation. Between days 20 and 24 of the experiment, these nymphs were incubated under controlled conditions at a temperature of 27°C and 85% relative humidity. This incubation facilitated the successful molting of the nymphs into adult ticks, which were subsequently used in the next phase of the study.

**Figure 3:**
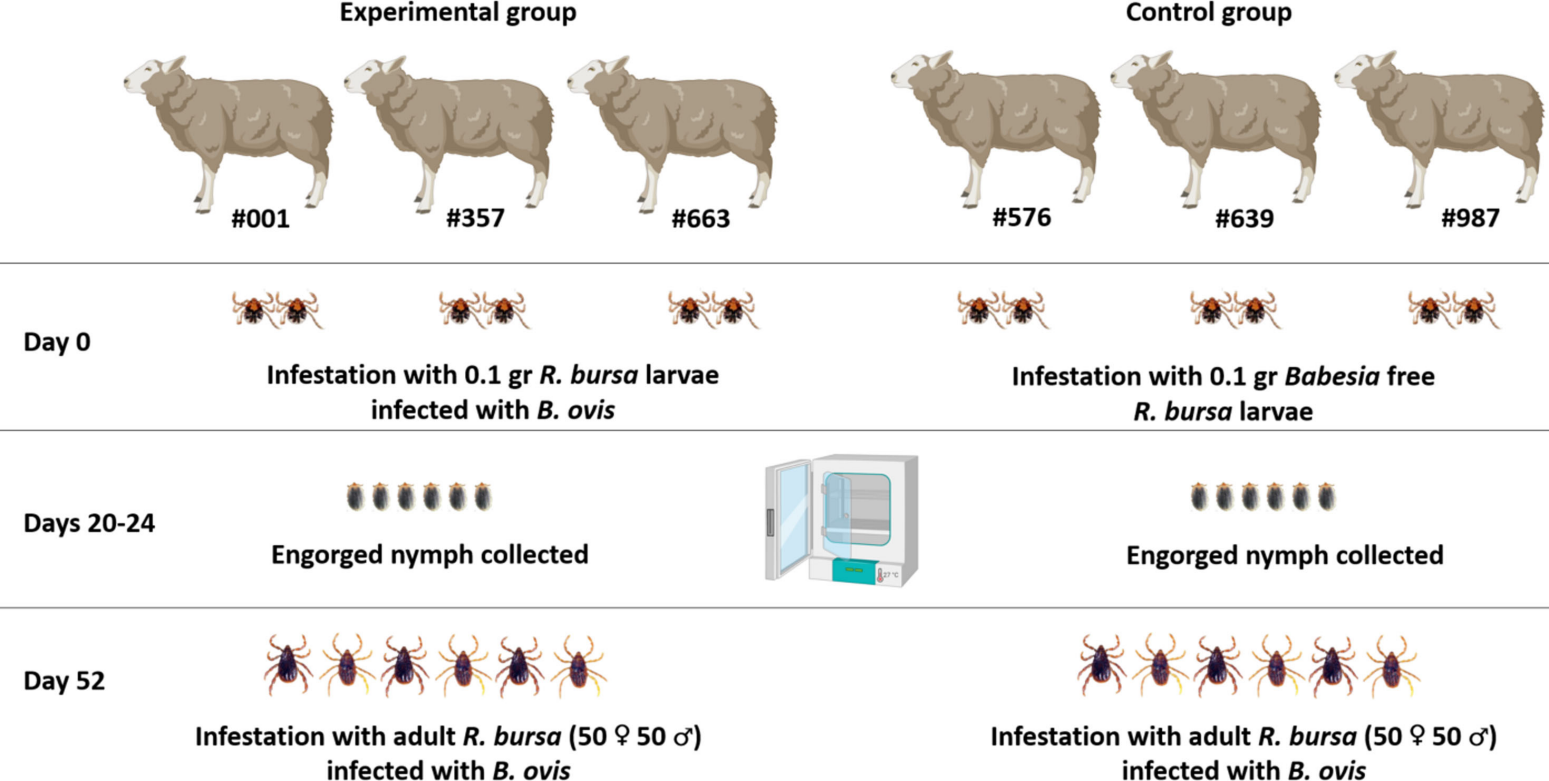
Experimental design for assessing the impact of *B*. *ovis*-infected *R. bursa* larvae on babesiosis severity in sheep. The experimental group (sheep #001, #357, #663) was infested with 0.1 g of *B*. *ovis*-infected *R. bursa* larvae on Day 0, while the control group (sheep #575, #639, #987) received an equivalent weight of *Babesia*-free *R. bursa* larvae. On Day 20-24, engorged nymphs were collected from both groups. Subsequently, On day 52, both groups were challenged with *B*. *ovis*-infected adult *R. bursa* ticks (50♂ and 50♀) to evaluate the clinical course of babesiosis. Figure was created using Biorender.com.

To confirm the presence of *B. ovis*, 30 adult ticks (15 females, 15 males) were collected from each sheep in the experimental group (#001, #357, #663). Ticks were organized into 10 pools per sheep, comprising 5 pools of females (3 female ticks per pool) and 5 pools of males (3 male ticks per pool). Each pool was analyzed individually by nPCR to verify infection status (Aktaş et al., 2005), and only positive pools were used for subsequent infestations (**Figure 3**).

The sheep were monitored daily for clinical signs of babesiosis, including fever, anemia, jaundice, and hemoglobinuria. Fever was measured using a digital rectal thermometer, while anemia was assessed by analyzing hematocrit (HCT) level using an automated hematology analyzer (Mindray BC-5000 Vet; Bio-Medical Electronics Co. Ltd., Shenzhen, China). Jaundice was evaluated visually by inspecting the mucous membranes for yellow discoloration, and hemoglobinuria was identified by observing the urine for dark red or brown pigmentation. Blood samples were collected in EDTA and serum tubes daily for molecular and serological analysis. Giemsa-stained blood smears were examined microscopically under a 100X oil immersion objective lens to identify the presence of parasitized erythrocytes, and the percentage of parasitized erythrocytes (PPE) was calculated based on at least 20 microscopic fields (Ozubek and Aktas, 2017).

### Genomic DNA extraction, PCR, and data analysis

Genomic DNA was extracted from both blood and tick samples using the PureLink™ Genomic DNA Mini Kit (Invitrogen, USA), following the manufacturer’s instructions. For blood samples, 200 µL of whole blood was processed according to the kit protocol to ensure efficient DNA isolation. For tick samples, female carcasses were bisected using a sterile scalpel after the oviposition period. One half of each carcass was placed into an eppendorf tube, crushed in liquid nitrogen to ensure thorough homogenization, and stored at -20°C until further processing. Approximately 500 larvae from each female tick were also selected, homogenized in liquid nitrogen, and stored under the same conditions. Genomic DNA was then isolated from these prepared samples, ensuring high-quality DNA suitable for downstream molecular analysis. Nested PCR assays were conducted for the detection of *Babesia*, *Theileria*, *Anaplasma*, and *Ehrlichia* species, as well as *B. ovis*, using previously established protocols (Georges et al., 2001; Bekker et al., 2002; Aktaş et al., 2005; Kawahara et al., 2006; Oosthuizen et al., 2008). For the first round of PCR, each reaction was prepared in a total volume of 25 μL, containing 2.5 μL of 10X PCR buffer (VitaTaq, Procomcure Biotech, Australia), 2.5 μL of each dNTP (1.25 mM), 0.1 μL of Taq DNA polymerase (5 U/μL), 0.5 μL of each outer primer specific to the target pathogen (20 pmol/μL), 2.5 μL of template DNA, and 16.4 μL of nuclease-free water. For the nested PCR, 1 μL of the first-round PCR product was used as the template, with the reaction mixture and cycling conditions consistent with the first round. Each PCR reaction included positive controls (genomic DNA of *B. ovis* GenBank accession number EF092454.1; *Anaplasma ovis* GenBank accession number MG642087.1) and negative controls (DNase/RNase-free water) to ensure the accuracy and reliability of the results. Amplifications were carried out in an automated DNA thermal cycler (Sensequest Labcycler Gradient, Göttingen, Germany). PCR products (10 μL) were electrophoresed on 1.4% agarose gels in TBE buffer for 30 minutes under a constant voltage, and the bands were visualized using the Quantum Vilber Lourmat Gel Imaging System (Marne-la-Vallée, France). Amplification bands corresponding to expected product sizes were clearly identified (**Supplementary Table 1**).

**Figure 4:**
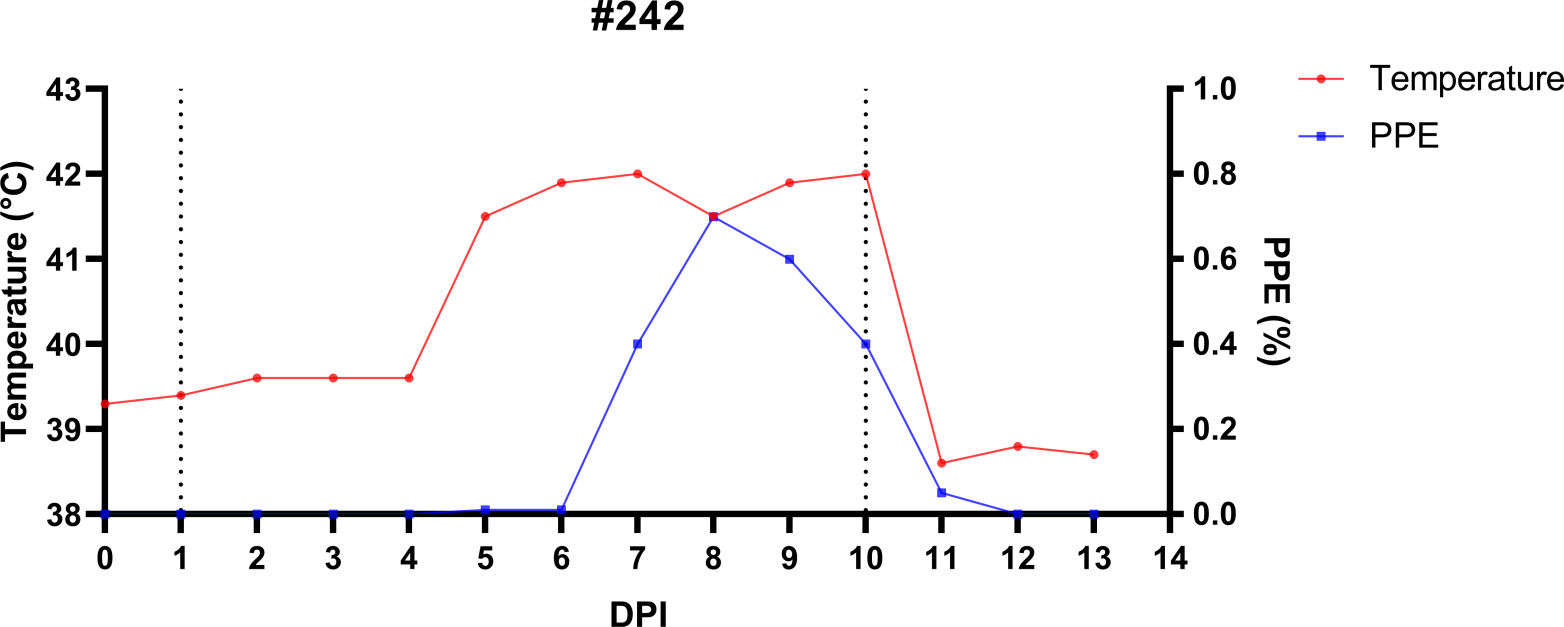
Temporal dynamics of infection and treatment in splenectomized sheep (#242) experimentally infected with *B*. *ovis* Alacakaya stabilate to produce *R. bursa* larvae infected with *B*. *ovis*. On day 1 post-infection (DPI 1), sheep #242 was infested with *Babesia*-free *R. bursa* female and male ticks. All engorged female ticks were collected on DPI 10 and left in the incubator to produce infected larvae. Post-collection, the sheep was treated with imidocarb dipropionate (1.2 mg/kg; IM).

**Figure 5:**
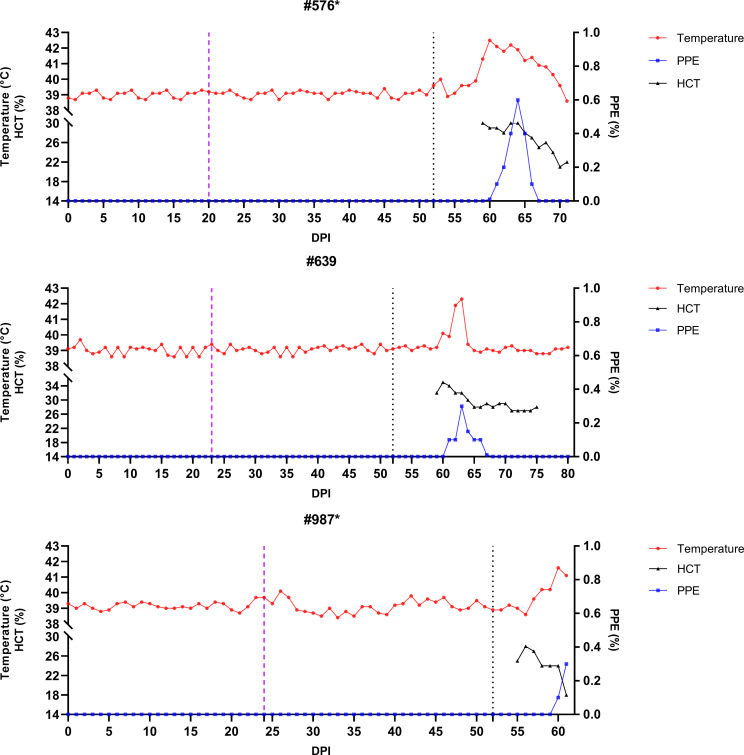
Monitoring of temperature, hematocrit (HCT), and parasitemia (PPE) levels in experimental group sheep (#576, #639, #987) after infestation with *Babesia*-free *R. bursa* larvae. Engorged nymphs were collected on Days 20–23 (purple dashed line), and adult *R. bursa* ticks infected with *B*. *ovis* were infested on Day 52 (black dashed line). Sheep marked with * succumbed to babesiosis during the study.

**Figure 6:**
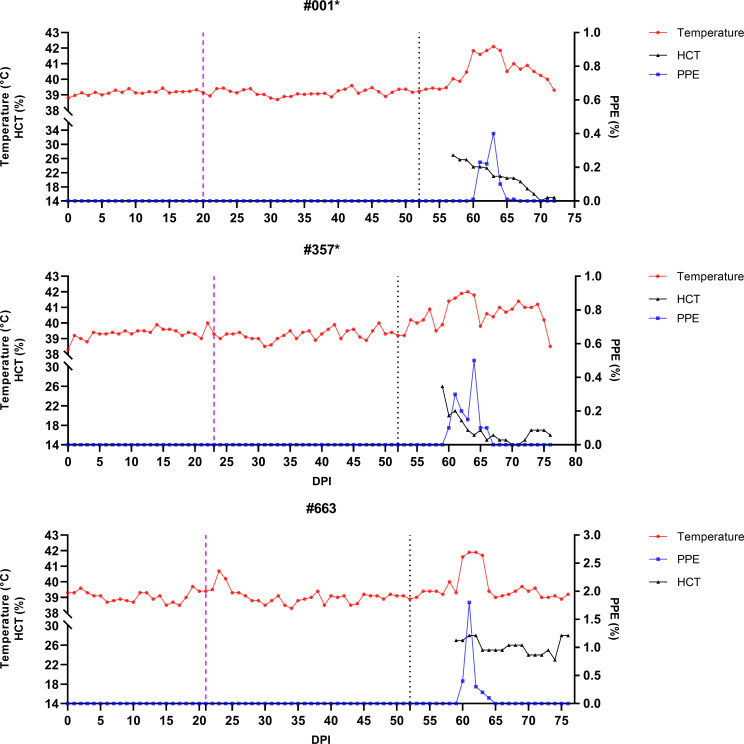
Monitoring of temperature, hematocrit (HCT), and parasitemia (PPE) levels in control group sheep (#576, #639, #987) after infestation with *Babesia*-free *R. bursa* larvae. Engorged nymphs were collected on Days 20–24 (purple dashed line), and adult *R. bursa* ticks infected with *B*. *ovis* were infested on Day 52 (black dashed line). Sheep marked with * succumbed to babesiosis during the study.

**Figure 7:**
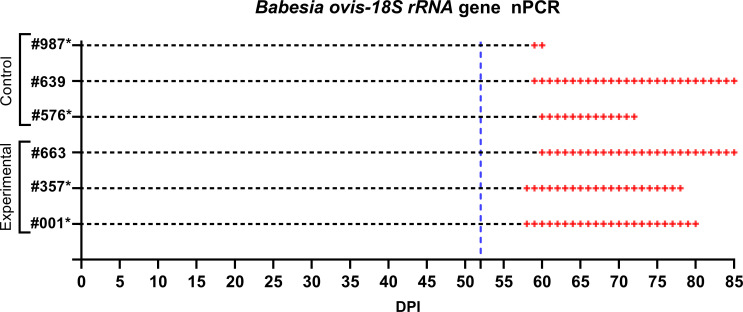
Monitoring of control and experimental group sheep from the start of larval infestation through to the end of the study for *B*. *ovis* detection via nPCR. Both groups were tested daily for *B*. *ovis* infection. The blue dashed line indicates the point at which the sheep were infested with adult *R. bursa* ticks infected with *B*. *ovis*. nPCR results are indicated as: **-**: nPCR negative, **+**: nPCR positive. Sheep marked with * succumbed to babesiosis during the study.

**Figure 8:**
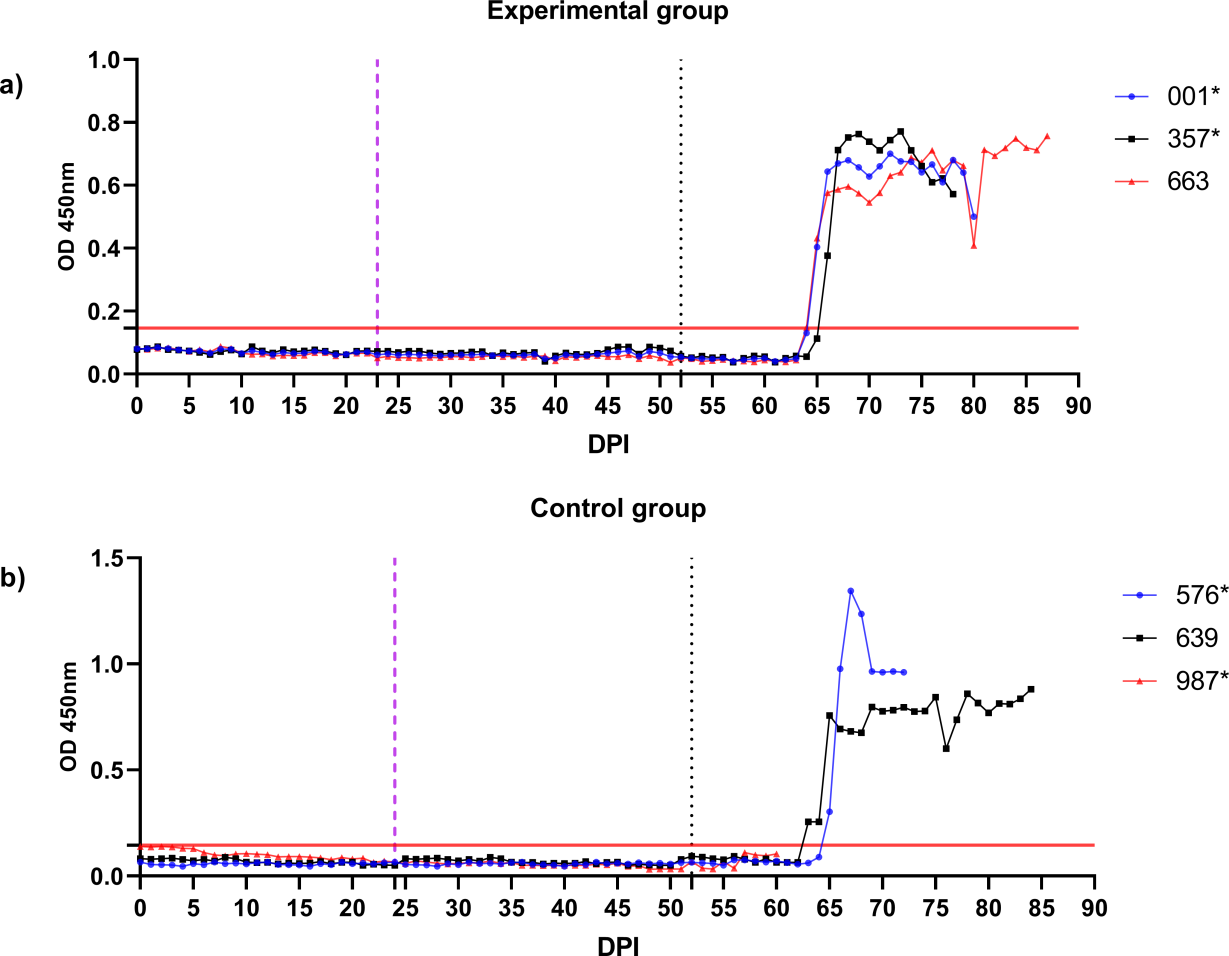
Monitoring of *B*. *ovis* BoSA1-specific antibody responses in control and experimental group sheep using iELISA. Antibody levels were measured periodically from the start of larval infestation through to the end of the study. Engorged nymphs were collected on Days 20–24 (purple dashed line), and adult *R. bursa* ticks infected with *B*. *ovis* were infested on Day 52 (black dashed line). The red line on the y-axis represents the cutoff value for the iELISA assay. Sheep marked with * succumbed to babesiosis during the study.

Descriptive statistics, including the calculation of mean values and standard deviations, were performed to analyze temperature, HCT, and parasitized erythrocyte percentage (PPE) data. Graphical representations, such as line charts and scatter plots (**Figures 4**–**8**), were generated using GraphPad Prism software version 8 (GraphPad Software, San Diego, CA).

## Results

### Acquisition of *Babesia ovis-*infected *Rhipicephalus bursa* larvae

The splenectomized sheep (#242) was monitored daily to track the progression of *B. ovis* infection and to produce infected *R. bursa* larvae. After the administration of the *B. ovis* Alacakaya stabilate, the sheep’s body temperature began to rise on day 5 post-infection (DPI 5), reaching a peak of 42°C on DPI 8. Concurrently, the percentage of parasitized erythrocytes (PPE) increased steadily, peaking at 0.8% on DPI 9, which marked the acute phase of the infection (**Figure 4**). During this period, clinical signs of babesiosis, including high fever, anemia, jaundice, and lethargy, were observed. These clinical manifestations were managed with the administration of 1.2 mg/kg imidocarb dipropionate (IMDP) on DPI 10, which helped alleviate symptoms and allowed for controlled recovery.

On DPI 10, all engorged female ticks were collected from the sheep and incubated under controlled conditions (27°C, 85% relative humidity). Larvae began to emerge within 30–34 days, and the emergence period lasted 7–14 days. Nested PCR confirmed that all engorged female ticks and their larvae were infected with *B. ovis* (**Supplementary Figure 1**). These larvae were subsequently used for the infestation of experimental sheep, forming a critical part of the study’s transmission model.

### Infestation outcomes and monitoring of experimental and control groups

Following the infestation of the experimental group with *B. ovis*-infected larvae and the control group with *Babesia*-free larvae, no clinical signs of babesiosis were observed in either group during the larval infestation period. Both molecular and serological analyses consistently showed negative results for *B. ovis* in all sheep during this stage (**Figures 5**, **6**). Engorged nymphs collected between DPI 12 and DPI 24 successfully molted into adult ticks within 18–29 days post-collection. Before the DPI 52 infestation with adult ticks, all adult ticks derived from the experimental group were tested in pools, and all pools were confirmed positive for *B. ovis* via nPCR. This confirmed the infection status of the larvae used in the initial infestation. On DPI 52, all sheep were infested with adult ticks derived from nymphs of the experimental group. This adult tick infestation marked the onset of clinical signs of babesiosis, with clear differences in disease progression between the experimental and control groups.

In the experimental group, clinical signs of babesiosis appeared within a prepatent period of 5 to 8 days following adult tick infestation. Sheep #001 and #357 developed severe disease, exhibiting sustained fever ranging from 39.4°C to 42°C for 10 to 11 days. Maximum parasitemia levels reached 0.4% and 0.5%, respectively, accompanied by severe anemia with HCT levels dropping to a minimum of 14%. Hemoglobinuria was also observed. Molecular detection using nested PCR (nPCR) confirmed the presence of *Babesia ovis* DNA by day 6 post-infestation (DPI) (**Figure 7**), while serological analysis using iELISA detected antibodies by DPI 12 to 13 (**Figure 8**). Despite supportive care, both sheep succumbed to the infection due to the severity of the disease. Sheep #663, also in the experimental group, displayed a shorter prepatent period of 5 DPI and reached the highest parasitemia observed in the study, at 1.8%. Although this sheep experienced fever and anemia, the clinical signs were notably less severe compared to Sheep #001 and #357. Fever persisted for 5 days, and HCT levels dropped to 24%, indicating moderate anemia. Molecular detection of *B. ovis* was achieved at DPI 8 (**Figure 7**), and antibodies were confirmed by iELISA at DPI 12 (**Figure 8**).

In the control group, clinical outcomes were similarly severe in two of the three sheep, but with notable differences in disease progression. Sheep #576 reached a parasitemia peak of 0.6% and exhibited clinical signs of severe anemia, fever, and hemoglobinuria. Molecular detection of *B. ovis* was achieved by DPI 8 (**Figure 7**), and antibodies were detected via iELISA at DPI 13 (**Figure 8**). Sheep #987 displayed the most rapid disease progression among all sheep, succumbing on the second day of parasitemia. This sheep had a maximum parasitemia of 0.3% and exhibited severe anemia and fever. However, antibodies were not detected by iELISA due to the rapid progression of the disease (**Figure 8**), which left insufficient time for a detectable immune response to develop. Sheep #639, the sole survivor in the control group, displayed milder clinical signs compared to the others. Maximum parasitemia in this sheep reached 0.3%, with fever persisting for 4 days. The HCT level dropped to 27%, indicating mild anemia. Molecular detection of *B. ovis* DNA occurred at DPI 7 (**Figure 7**), and antibodies were confirmed by iELISA at DPI 11 (**Figure 8**). This sheep demonstrated a less severe disease progression compared to Sheep #576 and #987.

## Discussion

This study sought to evaluate whether infestation with *B. ovis*-infected *R. bursa* larvae could lead to a milder form of babesiosis compared to sheep infested with larvae. Contrary to the hypothesis that infected larval infestation might induce a protective immune response against subsequent exposure to infected adult ticks, our findings conclusively demonstrated that *B. ovis*-infected larvae neither cause disease nor confer any protective effect.

Our results showed no evidence of *B. ovis* infection or clinical babesiosis following infestation with infected larvae, as confirmed by negative nPCR and iELISA results in all sheep. These findings align with previous studies, such as Erster et al. (2016), which established that larvae of *R. bursa* are incapable of transmitting *B. ovis*. Furthermore, no seroconversion or parasitemia was observed in the experimental group, even after prolonged monitoring up to day 52, further supporting the notion that larval stages do not play a role in the transmission dynamics of *B. ovis*. This contrasts with earlier suggestions by Yeruham et al. (1998), which proposed a delayed immune response following larval infestation. Our findings provide robust evidence against this hypothesis and suggest that any reported serological responses in previous studies might be attributed to differences in experimental conditions, diagnostic methods, or host variability. The inability of larvae to transmit *B. ovis* could reflect biological barriers such as insufficient pathogen replication, absence of salivary components required for transmission, or host-specific immune factors at the larval feeding stage.

In stark contrast to the larval stage, adult *R. bursa* ticks were confirmed to be the primary vectors for *B. ovis* transmission. Following infestation with *B. ovis*-infected adult ticks, all sheep developed clinical babesiosis, characterized by fever, anemia, and hemoglobinuria. No significant differences in disease severity were observed between the experimental and control groups. For instance, PPE levels peaked at 0.4%–0.5% in sheep #001 and #357 (experimental group) and at 0.6% in sheep #576 (control group). This indicates that prior exposure to infected larvae did not mitigate the severity of the disease caused by adult ticks. Interestingly, individual variability in disease progression was evident. Sheep #987 exhibited rapid disease progression, succumbing on the second day of parasitemia, whereas sheep #639 displayed a milder disease course with lower parasitemia levels and faster recovery. Although temporal differences in disease progression between groups were not observed in this study, further investigation into this aspect could help clarify whether prior larval infestation has subtle immunomodulatory effects that are not apparent in small sample sizes. These differences suggest that host-specific factors, such as genetic predisposition, immune competence, or other physiological traits, may influence the clinical outcome of *B. ovis* infection. Identifying these factors could provide valuable insights into host-pathogen interactions and inform selective breeding programs to enhance disease resistance in livestock.

The differential ability of tick life stages to transmit *Babesia* spp. reveals a fascinating interplay of biological, ecological, and epidemiological factors that shape parasite-vector-host dynamics. In our study, we observed that immature stages of *R. bursa*, particularly larvae, could acquire *B. ovis* but were unable to transmit the parasite effectively. This finding aligns with previous research highlighting stage-specific barriers in tick-borne pathogen transmission (Erster et al., 2016). Such stage-specific dynamics are not unique to *R. bursa* but are a common feature in the transmission cycles of various *Babesia* species, emphasizing the complex evolutionary adaptations of both the parasite and its vector. For instance, only larval stages of *Rhipicephalus microplus* transmit *Babesia bovis*, whereas transmission of *Babesia bigemina* is restricted to nymphs and adults (Dalgliesh and Stewart, 1983). This stage-specificity may be attributed to differences in host attachment preferences, feeding behavior, or immune responses encountered by ticks during their development. A contrasting example is *Babesia divergens*, which displays a broader transmission capacity, as all life stages larvae, nymphs, and adults can effectively transmit the parasite. Notably, engorging female ticks play a pivotal role in acquiring the infection, which is subsequently transmitted transovarially to their progeny, maintaining the parasite within tick populations and across hosts. This highlights a unique interplay between transstadial and transovarial transmission mechanisms in perpetuating *B. divergens* (Donnelly and Peirce, 1975; Lewis and Young, 1980). Similarly, *Babesia canis* demonstrates stage-specific transmission patterns with its vector, *Dermacentor reticulatus*. Adults are the primary transmitters due to their feeding preferences on canine hosts, while immature stages fail to transmit the pathogen effectively. The inability of immature ticks to act as competent vectors may reflect barriers in parasite acquisition during earlier stages or limitations in pathogen development within the vector (Ristic, 1988). These observations underscore the importance of studying the immune responses elicited by immature tick feeding. Investigating whether larval or nymphal feeding primes the host’s immune system in ways that could influence subsequent pathogen transmission remains an important avenue for future research.

To summarize, this study confirms that the transmission of *B. ovis* is restricted to adult *R. bursa* ticks, while larvae, although capable of acquiring the parasite, do not induce clinical or immunological effects in their hosts. These findings reaffirm the critical role of adult ticks as the primary vectors for *B. ovis*, consistent with previous studies. However, a key insight from this study is that larval feeding does not influence the clinical outcomes of subsequent infections with adult ticks, suggesting that subclinical infections or milder disease courses observed in natural settings are unlikely to be related to larval feeding. This finding highlights the importance of focusing control strategies on adult tick populations, particularly during periods of peak activity, to effectively mitigate the spread of ovine babesiosis. Beyond the implications for *B. ovis*, these findings raise broader questions about the potential epidemiological role of immature tick stages in other tick-borne disease systems. Although immature ticks may not directly transmit pathogens, they can serve as reservoirs, maintaining infections within tick populations through transstadial transmission. Such dynamics have been observed in *B. bovis* and *B. bigemina*, where immature stages acquire the pathogen and ensure its persistence until competent stages emerge. Future studies should explore the molecular and physiological mechanisms underlying the inability of immature ticks to transmit *B. ovis*. Investigating whether larval and nymphal feeding induces subtle immunomodulatory effects or primes host immunity could reveal important host-pathogen-vector interactions. Moreover, the application of longitudinal immune profiling and gene expression analyses could clarify whether prior larval infestation affects the host’s susceptibility to subsequent infections. Investigating the barriers that prevent transmission in immature stages, such as developmental or molecular limitations, could uncover new insights into the complex life cycles of *Babesia* parasites and their vectors. By exploring these dynamics, future research could identify novel strategies for disrupting the parasite’s life cycle at multiple stages of the tick’s development, enhancing vector control efforts.

In conclusion, our study demonstrates that the transmission of *B. ovis* occurs predominantly through adult *R. bursa*, with the larval stage playing no significant role in the transmission cycle. Furthermore, exposure to *B. ovis* infected larvae does not result in milder disease outcomes or provide any protective immunity against subsequent infections by adult ticks. These findings emphasize the importance of prioritizing the control of adult *R. bursa* populations to effectively manage the spread of ovine babesiosis. Future research should aim to uncover the molecular and physiological mechanisms that limit the ability of immature ticks to transmit *B. ovis*. Such insights could pave the way for innovative strategies to disrupt the parasite’s life cycle and improve vector management practices. Additionally, comparative studies across different *Babesia* species and their vectors could offer valuable perspectives on the evolutionary and ecological factors influencing stage-specific transmission dynamics.

## Data Availability

The raw data supporting the conclusions of this article will be made available by the authors, without undue reservation.
