# Supplementary material for: The impact of Babesia ovis-infected Rhipicephalus bursa larvae on the severity of babesiosis in sheep
Source: Front Cell Infect Microbiol. 2025 Feb 20;15:1544775. doi: 10.3389/fcimb.2025.1544775 (PMC11882592; doi:10.3389/fcimb.2025.1544775)
Supplement: Supplementary file 1 [file Table1.docx]

**Supplementary Table 1:** Primers and sequences used in this study for the detection of tick-borne pathogens

| **Tick-borne pathogens** |  | **Target gene** | **Primer** | **Primer sequence (5′–‘3)** | **Product size (bp)** | **Reference** |
| --- | --- | --- | --- | --- | --- | --- |
|  | 1^st^ reaction | *16S rDNA* | Ec9 | TACCTTGTTACGACTT | 1462 | Kawahara et al., 2006 |
|  |  |  | Ec12A | TGATCCTGGCTCAGAACGAACG |  |  |
| *Anaplasma* / *Ehrlichia* |  |  |  |  |  |  |
|  | nPCR |  | 16S8FE | GGAATTCAGAGTTGGATC(A/C)TGG(C/T)TCAG | 492-498 | Bekker et al., 2002 |
|  |  |  | BGA1B | CGGGATCCCGAGTTTGCCGGGACTT(C/T)TTCT |  |  |
|  | 1^st^ reaction | *18S rDNA* | Nbab1F | AAGCCATGCATGTCTAAGTATAAGCTTTT | 1600 | Oosthuizen et al., 2008 |
|  |  |  | Nbab1R | CTTCTCCTTCCTTTAAGTGATAAGGTTCAC |  |  |
| *Babesia* /*Theileria* |  |  |  |  |  |  |
|  | nPCR |  | RLBF2 | GACACAGGGAGGTAGTGACAAG | 390-430 | Georges et al., 2001 |
|  |  |  | RLBR2 | CTAAGAATTTCACCTCTGACAGT |  |  |
|  | 1^st^ reaction |  | Nbab1F | AAGCCATGCATGTCTAAGTATAAGCTTTT | 1600 | Oosthuizen et al., 2008 |
|  |  |  | Nbab1R | CTTCTCCTTCCTTTAAGTGATAAGGTTCAC |  |  |
| *Babesia ovis* |  | *18S rDNA* |  |  |  |  |
|  | nPCR |  | BboF | TGGGCAGGACCTTGGTTCTTCT | 549 | Aktas et al. 2005 |
|  |  |  | BboR | CCGCGTAGCGCCGGCTAAATA |  |  |
