# Supplementary material for: The impact of Babesia ovis-infected Rhipicephalus bursa larvae on the severity of babesiosis in sheep
Source: Front Cell Infect Microbiol. 2025 Feb 20;15:1544775. doi: 10.3389/fcimb.2025.1544775 (PMC11882592; doi:10.3389/fcimb.2025.1544775)
Supplement: Supplementary file 2 [file DataSheet1.docx]

Supplementary Material





**Supplementary Figure 1.** Gel electrophoresis results confirm the presence of *B. ovis* in engorged female ticks and their larvae. The top panel shows nPCR results obtained with BboF-BboR primers, while the bottom panel displays PCR results using Nbab1F-Nbab1R primers. Lane assignments are as follows: 1: Engorged female-1, 2: Larvae-1, 3: Engorged female-2, 4: Larvae-2, 5: Engorged female-3, 6: Larvae-3, 7: Engorged female-4, 8: Larvae-4, 9: Engorged female-5, 10: Larvae-5, 11: Engorged female-6, 12: Larvae-6, 13: Engorged female-7, 14: Larvae-7, 15: Engorged female-8, 16: Larvae-8, 17: Engorged female-9, 18: Larvae-9, 19: *Theileria annulata* (AY508463) positive control, 20: *Babesia ovis* (PP973837.1) positive control, and 21: Distilled water negative control. A molecular size marker (M) is included on the left, with 500 bp and 1200 bp bands indicated.
